# Semiconductor optical amplifier-based laser system for cold-atom sensors

**DOI:** 10.1140/epjqt/s40507-025-00348-z

**Published:** 2025-04-10

**Authors:** Eric Kittlaus, Jonathon Hunacek, Mahmood Bagheri, Hani Nejadriahi, Mehdi Langlois, Sheng-wey Chiow, Nan Yu, Siamak Forouhar

**Affiliations:** Jet Propulsion Laboratory, California Institute of Technology, Pasadena, CA 91009 USA

**Keywords:** Semiconductor optical amplifiers, Atom interferometry, Flyable systems

## Abstract

Precise control of atomic systems has led to an array of emerging ‘quantum’ sensor concepts ranging from Rydberg-atom RF-electric probes to cold-atom interferometer gravimeters. Looking forward, the potential impact of these technologies hinges on their capability to be adapted from laboratory-scale experiments to compact and low-power field-deployable instruments. However, existing setups typically require a bulky and power-hungry laser and optics system (LOS) to prepare, control, and interrogate the relevant atomic system using a variety of frequency-referenced and rapidly reconfigurable laser beams. In this work, we investigate the feasibility of using semiconductor optical amplifiers (SOAs) to replace high-power pump lasers and acousto-optic modulators within a simple atom cooling apparatus, looking forward to the ultimate goal of a space-deployable atom interferometer. We find that existing off-the-shelf SOA components operating at relevant wavelengths for Cs and Rb atom cooling (852 and 780 nm, respectively) are able to permit an attractive combination of rapid (sub-microsecond), high extinction ratio (>60-65 dB) switching while acting as power boosters prior to the atom physics package. These attributes enable a radically different, power-efficient approach to LOS design, reducing or eliminating the need for Watt-class laser amplifiers that are unsuitable for flight deployment. Building on these results, we construct a simple and compact all-semiconductor laser/amplifier LOS for atom cooling that is integrated with custom path-to-flight drive electronics. Up to 125 mW of total optical power is delivered to six fiber-coupled channels for magneto-optical-trap-based cooling of a cloud of neutral Cs atoms. The entire LOS, including reference and cooling laser subsystems and control electronics, occupies a volume of 20×20×15 cm and totals DC power consumption of around 13.5 W, and is designed in a modular format so that additional hardware for synthesizing atom interferometry beams may be added through future development efforts. These results indicate the utility of all-semiconductor laser systems for future low-power flyable atom-based sensor instruments.

## Introduction

In recent years, quantum measurement techniques based on ultracold atoms have advanced significantly for metrology and sensing applications ranging from ultra-precise clocks [1–5] to interferometer-based accelerometers and gravimeters [6–11]. Notably, while atom interferometer gravimeters are already widely investigated for terrestrial applications, longer interrogation times, and hence dramatically improved sensitivity, are realizable in microgravity environments [12], opening the possibility for high-sensitivity gravity cartography from orbit [8, 13, 14]. As a result, cold-atom interferometry offers potential breakthrough performance of next-generation satellite-based remote gravity mapping for Earth science applications [15].

To date, cold-atom sensors have generally operated as large, meter-scale experiments with substantial supporting laboratory hardware, although considerable progress has been made toward miniaturized reference cells and supporting microwave interfaces [16, 17]. While compact atomic clocks are now commercially available [18, 19], miniaturization of sophisticated sensors based on laser-cooled atoms remains a key goal [20–22]. Each of these instruments requires a complex laser and optics system (LOS) for atomic state preparation, control, and interrogation. To this end, there have been substantial efforts toward integration of high-performance lasers and detectors [23–28]. Nonetheless, the stringent requirements of frequency-agile control, high-speed switching, and sufficient optical power delivered to the atom physics package make it challenging to reduce system size, weight, and power (SWaP). Optical insertion losses through numerous successive switches, modulators, isolators, and passive components typically necessitate the use of high-power lasers or amplifiers, which can both complicate thermal management and pose a risk for degradation due to high electrical current densities.

Current LOS designs for field-deployable atom interferometers typically utilize one of two approaches. The first is based on frequency-doubled laser systems, which are particularly used for ^87^Rb or ^40^K species that have optical transitions around 780 nm and 767 nm, respectively [29–31]. In this case, mature telecom-wavelength lasers, combined with high power erbium-doped fiber amplifiers, can be used in conjunction with poled nonlinear crystals to generate high power signals with narrow optical linewidth around this specific wavelength band. The second approach uses the combination of low-noise lasers, such as external cavity diode lasers (ECDL), with high power tapered amplifiers (TA) in a so-called master oscillator power amplifier (MOPA) configuration [13, 32]. In both cases, high power laser oscillators are used to distribute signals to an array of down-stream components. The use of these sources operating at high (Ampere-level) drive currents leads both to high power consumption, and may raise concerns about instrument lifetime, particularly looking forward to remote operation in harsh environments. As a point of reference, current state-of-the-art for terrestrial instruments [10, 31], as well as technology demonstration missions such as NASA’s Cold Atom Lab operated aboard the International Space Station [13, 14], are large (∼1 m scale) instruments drawing hundreds of Watts of power under typical conditions.

Beyond the requirement for high optical powers and rapid frequency agility, switching and pulse shaping requirements are particularly stringent for careful preparation and interrogation of cold atom sensors. An ideal optical switch in this case should offer high on-off extinction ratio (typically > 60 dB) so that beams in the ‘off’ state do not negatively impact the atomic ensemble, while power modulation and switching at sub-μs-timescales is desired for shaping of pulses used to implement various interferometery schemes. Acousto-optic modulators (AOMs) are usually the switch of choice to meet these requirements due to their stability and fast switching times, and for their capability for fine frequency and amplitude control. However, bulk-optic AOMs tend to be extremely power hungry—typically requiring 0.2-2 W RF drives generated using inefficient RF power amplifiers, leading to power consumption of several Watts per individual component. Other switching technologies, such as microelectromechanical-optical switches or electro-optic switches typically do not offer sufficient performance alone. While combinations of slow (> millisecond) mechanical switches with fast but low-extinction (<30 dB on/off) electro-optic modulators can be realized [13], this increases the number of component types based on different material platforms that must be qualified for operation in harsh environments. The lack of power-efficient techniques for high-fidelity switching and amplitude modulation poses an unexpected barrier to size and power reduction for complex atom interferometer systems which may require dozens of such components.

In this paper, we investigate semiconductor optical amplifiers (SOAs) as a potential tool for developing power-efficient cold-atom sensors targeted for space applications. In our implementation, SOAs act as both power boosters immediately prior to the atom physics package, and as high-performance optical switches that offer extremely fast (nanosecond-scale) switching speeds, high on-off extinction ratio (>60-65 dB), and solid-state operation. Combining a bank of multiple SOAs with semiconductor seed lasers, detectors, passive optics, and custom path-to-flight electronics, we implement a novel ‘all-semiconductor’ approach to the construction of a compact ($20 \times 20\times 15$ cm) and low power (13.5 W DC) LOS operating around 852 nm for Cs atom cooling. Looking forward to applications in science-grade sensors, we investigate the performance of SOAs in terms of switching dynamics, noise, stability, and reliability under pulsed operation as would be needed for long-term operation of a remote cold atom interferometer gravimeter. To address power fluctuations observed through test of an array of SOA components, we implement closed-loop output power control across multiple channels with $>10\times $ improvement in short and long-term stability compared to free-running operation, even during implementation of pulse sequencing. In a simple laboratory experiment, we achieve cooling of a cloud of ∼10^6^ Cs atoms to demonstrate proof-of-concept operation. These results can be generalized to atomic species that require operation at other wavelengths from near-ultraviolet to infrared where semiconductor gain chips are available. Looking forward, further technology development may permit this approach to implement all necessary functionalities for fieldable atom interferometry or other cold-atom sensor architectures.

## Results

The objective of this work is to investigate approaches to the development of a laser and optics system (LOS) for atom interferometry with radically reduced size and power consumption as compared to existing systems. Such an LOS is ultimately intended for deployment in a low-power, Earth-orbiting gravity gradiometer which conducts differential gravity measurements using two atom interferometers separated by ∼1 m baseline [33]. More generally, for cold-atom sensors intended for field or spaceborne deployment, it is important that all components and supporting electronics have a clear path to flight qualification. For our notional design, we target operation on the D2 line (852 nm) of ^133^Cs atoms. This choice is intended to coordinate with ongoing instrument development efforts at NASA-JPL, while also demonstrating operation at a wavelength not readily reachable with existing architectures based on optical frequency doubling to ∼780 nm. The structure of this paper is as follows: we begin by describing the general architecture of the SOA-based laser system, and show its operation within a laboratory atom cooling experiment. Next, we investigate in detail the performance of constituent SOA components to establish general operation parameters and motivate the implementation of active power stabilization in our LOS to correct for short- and long-term output power drifts on a channel-by-channel basis. Additional tests of gain, extinction ratio, and added noise for the commercial SOA devices utilized are included in the Appendices for reference.

### SOA-based atom cooling

**Figure 1:**
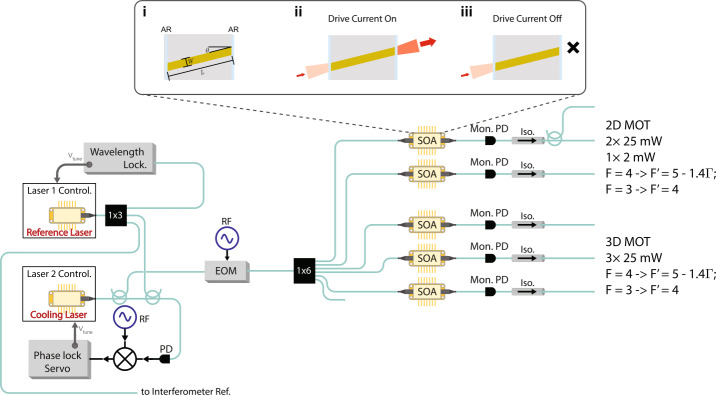
Block diagram of the SOA-based system for cooling of Cs atoms. A ‘Reference’ laser is locked to a hyperfine transition of neutral Cs atoms in a vapor cell via saturation absorption spectroscopy. A second ‘Cooling’ laser is offset-locked relative to the reference laser using an optical phase-locked loop and current feedback. SOAs with monitor photodiodes (see Sect. 2.4) serve as power boosters and amplitude modulators prior to the physics package consisting of 2D and 3D magneto-optical traps. Subplots i-iii depict the structure and operation scheme of a typical SOA chip: An angled waveguide is fabricated from a semiconductor heterostructure so that the guided mode has significant overlap with the active region, and is tilted relative to normal (typically 5-10 degrees) and anti-reflection coated to mitigate reflections that could lead to feedback. (ii-iii) When current is applied across the active region, input signals are amplified via optical transitions of excited carriers. When no current is present, input signals are strongly absorbed.

To evaluate the practical viability of SOAs for use within cold atom experiments, and demonstrate the power benefit of an SOA-based architecture, we designed a prototype system to implement the necessary functions for cooling of a cloud of Cs atoms, as shown in Fig. 1. The main operation principle for optical signal synthesis is based on a combination of laser frequency-referencing and agility of the low-power source lasers, combined with high-extinction ratio switching and amplitude control in each SOA component. All of the SOA devices tested through our investigation utilize a similar designs to that depicted in the inset Fig. 1.i: an optical ridge waveguide of width *W* and length *L* is designed so that the guided mode has some overlap with an active region, usually a compound semiconductor structure, which produces optical gain when electrically pumped. The waveguide itself is tilted by an angle *θ* with respect to normal or otherwise bent to reduce end-facet reflections, and anti-reflective coatings (AR) on the chip facets are also employed. These modifications are used to reduce reflections to typical levels of <10^−4^ to prevent lasing. When drive current is turned on, input light to the SOA is amplified, while when no current is applied, light is strongly absorbed in the SOA. Tuning the drive current allows precise control of the output power from each optical channel.

As depicted in the simplified block diagram (lower part of Fig. 1), the system incorporates a wavelength-stabilized ‘Reference’ laser that is locked to an atomic hyperfine transition (on the Cs D2 line in this case) using saturation absorption spectroscopy of a compact vapor cell. A second ‘Cooling’ laser is stabilized at an electrically-controlled offset frequency using an optical phase-locked loop, while a third path will route part of the Reference laser light to a future add-on board that will implement optical and electronic components to synthesize beam-splitter pulses and transport beams for atom interferometry experiments. An electro-optic phase modulator (EOM, EOspace, Inc.) is inserted in the cooling laser path to shift a small fraction of light (few %) to the repump frequency. Alternately, this EOM may be placed in a single one of the magneto-optical trap channels, or eliminated and replaced by a fraction of light from the reference laser if its center frequency is chosen to be identical to the repump frequency, depending on the desired configuration. Regardless, the placement of the EOM is somewhat flexible, as the mW-level maximum optical power handling of typical LiNbO_3_ modulators at these wavelengths does not pose an issue in this architecture. This results from the relatively low seed powers (∼0.1 mW) required to saturate each SOA. After passing through the EOM, the cooling signal split through a 1×6 planar lightwave circuit splitter (PLC Connections, Inc.), and is distributed to 5 SOAs which are used to synthesize signals fed to 2D and 3D MOTs for laser cooling.

This system is configured for Cs atom cooling using commercial 852 nm SOAs (Superlum SOA-372-DBUT-PM) and Reference and Cooling DBR lasers (Photodigm; measured integral linewidth ∼150 kHz around 852 nm). Each SOA’s current is tuned to provide 10-25 mW maximum output power at its respective inline monitor photodiode (Mon. PD; OZ Optics OPM-11-850) which can be used to implement active power control and fine-tuning as described in Sect. 2.4. Thereafter, light is directed through optical isolators and to the physics package, both of which are currently external to the LOS. Polarization maintaining fiber (PM780-HP) is used throughout, and typical measured polarization extinction ratios at the LOS output are >20 dB. Currently, at the board output, these connections to isolators and the physics package are made using physical FC/APC connectors for ease of reconfigurability–with small core fibers used for these wavelengths, this leads to a non-negligible source of loss due to core misalignment. Combined with ∼0.8 dB losses through each optical isolator (OZ Optics FOI-11-11-850), a maximum of 15 mW is delivered to each MOT channel of the physics package (total 45 mW for 3D-MOT using three SOAs). To achieve higher total output power, the SOAs could be directly replaced with SOAs designed for higher power operation–one such part (Superlum B850.30.40P, output >40 mW) has recently become commercially available.

**Figure 2:**
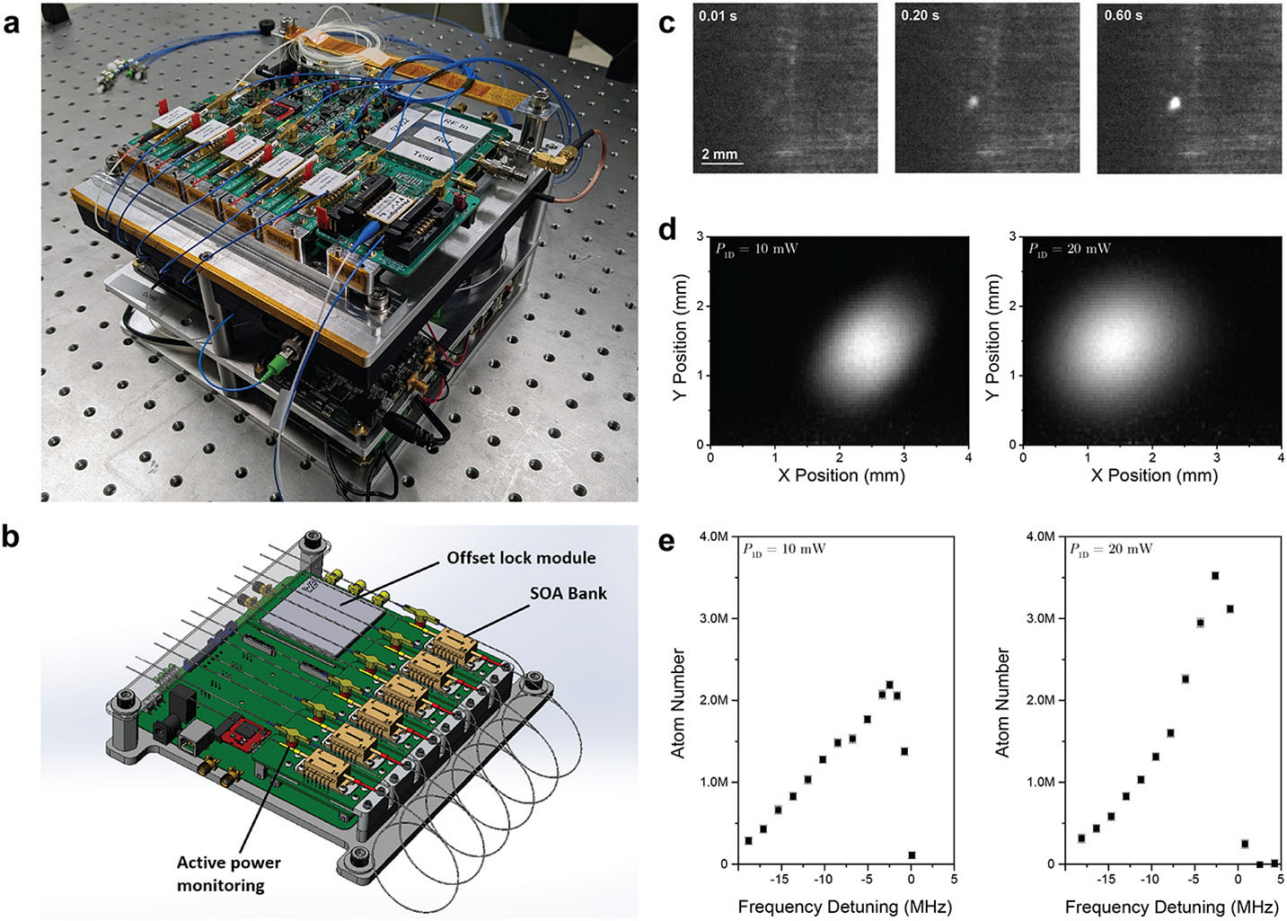
SOA-based laser system and demonstration of atom cooling. (a) shows a photograph of the assembled prototype system. This system includes custom drive and control electronics for SOAs and offset locking between reference and cooling lasers (top), and reference laser locking hardware and electronics underneath. (b) depicts an engineering drawing of the custom drive board used for active power monitoring and control of a bank of SOAs. (c) shows photographs of the formation of a cloud of cooled Cs atoms generated using this laser system, as revealed by laser-induced fluorescence. (d) plots more detailed photographs of the Cs cloud under two different nominal injection powers, while (e) plots the estimated atom number as a function of MOT-beam frequency detuning from resonance, showing the fine frequency tuning capability of the optical phase-locked loop, from [34]. The left and right panels correspond to optical power per axis of 10 mW and 20 mW, respectively, corresponding to total powers of 30 and 60 mW. Error bars represent 1 standard error of mean, though systematic errors in estimated absolute atom number are necessarily larger.

The assembled prototype LOS is shown in Fig. 2a, and utilizes a combination of commercial and custom in-house components. All electronics are ultimately intended to be replaced by custom flight-qualified versions, although some commercial boards are used for the Reference laser subsystem for rapid prototyping. The Reference laser itself is locked to a miniaturized Cs vapor cell wrapped in a Helmholtz coil (MOGLabs) that is directly modulated to synthesize the error signal for a saturation absorption spectroscopy absolute frequency lock at the ∼ kHz level. Automation during turn-on, as well as the locking feedback loop itself, is implemented using a commercial FPGA module (RedPitaya) and Linien package [35]. All of the Cooling laser operations, including laser and SOA drivers and thermal control for each, and offset locking including frequency tunability, are implemented using a custom electronics board (Fig. 2b). This same board implements active power control with the inline photodiodes and their bias circuits integrated with the board, as well as all electronics for independent control of each SOA. Lasers and SOAs are currently mounted to this control board using daughter boards for flexibility, but ultimately a bank of many SOAs could in principle be implemented in a micro-optic package to further reduce footprint. A module which provides all the electronics for the optical phase-locked loop (OPLL) also directly interfaces with the board, and can be removed or replaced for flexibility during development. All individual electronic components chosen are either flight heritage or have path-to-flight analogues which can be implemented in later revisions. Existing commercial electronics used to drive the Reference Laser (Koheron CTL200) and FPGA will be implemented in the same architecture, but our efforts to-date have focused on control of the Cooling laser system and SOAs where maximum benefit is realized from this initial hardware specialization. Full details of the system-level design and electronics configuration are reported separately, see Ref. [34].

For initial testing, the setup was transported to JPL’s Quantum Sciences & Technology Laboratory where its 3D-MOT outputs were connected to a functioning cold atom experiment setup: the commercial Cs atom physics package used (ColdQuanta, Inc.) consists of three pairs of Helmholtz coils surrounding a Cs vapor cell. Each MOT beam is injected along one of the three orthogonal axes (one of which is aligned to the horizontal MOT coil) through fiber collimators which output 7.8 mm diameter beams. Each of the 3 beams is retro-reflected for Doppler cooling. During a programmable sequence of pulses or power steps, the frequency of the beams is red-detuned from the F=4→F′=5 optical transition by around 1.4Γ (∼7.3 MHz, where Γ is the transition natural linewidth), while a small amount of light (few percent) is shifted to the F=3→F′=4 transition frequency (around +9.043 GHz away) using an electro-optic phase modulator to serve as repump beams. (The other modulation sideband at −9.043 GHz is also present, but is not resonant with the relevant atomic transitions.) When the SOAs are switched on, formation of a cloud of cooled Cs atoms is observed via laser induced fluorescence (Fig. 2c-d), showing proof-of-concept atom cooling with this simple architecture. The estimated lower bound on atom temperature is inferred by the Doppler cooling limit, $T_{\mathrm{D}} = \hbar \Gamma / 2 k_{\mathrm{B}}$, to >125 μK.

Frequency tunability of the cooling beams is achieved by stepping the microwave frequency used to set the optical phase-locked loop offset. As the 3D-MOT beams are stepped through the atomic resonance (Fig. 2(e), formation of a cloud of cooled atoms is observed, up to a peak atom number of 3.6 million in the observed cloud via integration of the fluorescence image. Ultimately, the 3D-MOT is intended to be fed by an auxiliary 2D-MOT. While the LOS provides the necessary optical beams for operation of both MOTs, at the time of test this functionality was not available to the physics package, limiting the cloud population to this relatively small number from free capture. Looking forward, implementation of optical molasses should be able to reduce atom temperature to <5 μK, but was not possible with the current physics package implementation due to the inability to generate a zero-magnetic-field state. Modifications to the physics package, as well as augmentation of the LOS to implement additional beams for transport and Bragg pulses for atom interferometry demonstrations, are ongoing (see Discussion).

Through these tests, the total DC power consumption for the cooling laser subsystem, including OPLL, is around 4 W in standby and 5.5 W with all SOAs tuned to max operating current. The Reference laser subsystem draws around 8 W, most of which (5 W) is consumed by the commercial FPGA module. All components are implemented in a footprint occupying approximately 20×20×15 cm, except for fiber management and isolators. We anticipate that these size and power requirements can be decreased further with hardware specialization–at present, however, these subsystems were designed to maximize flexibility during system development and test. Our general approach can be generalized to other wavelengths simply by swapping of optical components–for example, the system is also operable around 780 nm for Rb atoms by swapping out the reference cell, lasers, and amplifiers. Initial tests of 780 nm SOAs and DBR lasers in the same system are ongoing and have allowed us to compare performance characteristics between lasers and SOAs at these two wavelengths.

This demonstration shows that an all-semiconductor laser and amplifier system can permit atom cooling while eliminating the need for additional switch components or high-power lasers/MOPAs. For a future science-grade sensor instrument, performance requirements will be significantly stricter and require enhanced functionality. Keeping these requirements for a fieldable instrument in mind, we next investigate salient characteristics of SOA operation including phase-sensitive measurements of temporal dynamics and added noise. Additional tests carried out during LOS development included investigation of switching speed, high-extinction-ratio switching over a wide range of operation conditions, and initial tests of long-term reliability under pulsed operation, and are included in the Appendices for reference.

### Temporal dynamics

**Figure 3:**
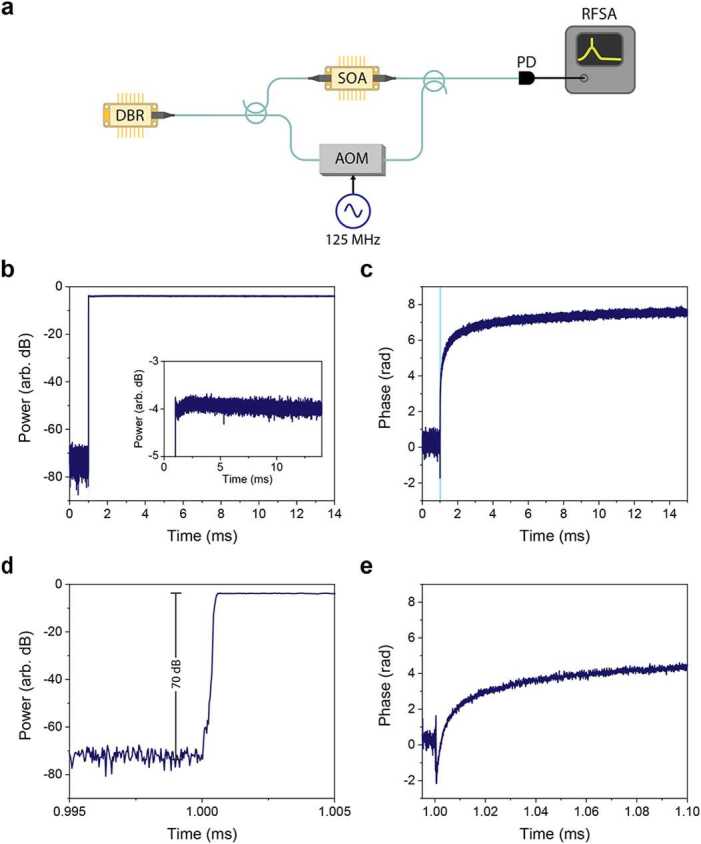
Temporal dynamics of SOA switching revealed by heterodyne measurements. (a) diagram of phase-sensitive measurement system. A DBR seed laser is split in two paths. In the top path, it is sent through the SOA under test, which is switched on/off using a pulsed driver. In the bottom path, light is frequency shifted using an acousto-optic modulator (AOM) to form a local oscillator for heterodyne detection. These two signals are then combined on a fast photodiode and their resulting beat-note is digitized using a RF signal analyzer. (b) plots the measured signal power over time, showing high extinction ratio switching, while an inset shows a zoomed-in plot revealing small fluctuations over the first few milliseconds after turn-on. (c) plots the measured optical phase over this same time period, revealing gradual shift of the optical phase after turn-on, likely due to thermalization over this time period. (d) and (e) show zoomed-in versions of the same data around the time where the SOA is switched on (highlighted blue regions of the above plots).

To study the behavior of SOA devices across relevant timescales for cold atom measurements, we implemented a phase-sensitive heterodyne detection scheme. As diagrammed in Fig. 3a, light from a seed laser (DBR) is split between the SOA under test, and a reference arm where it is frequency shifted by +125 MHz using an acousto-optic modulator. Light from both paths is combined on a fast photodiode, resulting in a radiofrequency beat-note at the heterodyne frequency with power proportional to the optical beat-note power, and which can be directly digitized using an RF signal analyzer (Keysight N9030B). Notably, this heterodyne measurement approach preserves information of both amplitude and relative frequency (phase) between the two paths as drive current of the SOA under test is pulsed, and allows rapid, high-dynamic-range measurement in the microwave domain. For the tests plotted in Fig. 3, square-wave current pulses of 120 mA were used for the 852 nm device. Tests repeated for several different commercial devices tested at either 780 nm (Innolume SOA-780-20-YY-30dB, Thorlabs BOA780P) or 852 nm (Superlum SOA-372-DBUT-PM) with drive currents around 100-200 mA observed qualitatively similar behaviors and are not shown for brevity. Through these studies, we intended to represent typical performance for commercial fiber-pigtailed semiconductor optical amplifier devices for Rb- or Cs-based atom interferometer instruments.

SOAs can produce fast, nanosecond-scale switching due to short carrier lifetimes (see Appendix A, for example). Our phase-sensitive tests of longer pulses studied over μs-ms timescales relevant for atom cooling (Fig. 3b-e), reveal an array of complex temporal dynamics. While, through all tests, on-off extinction of 60-70 dB is achieved (for full details see Appendix B), fluctuations in amplitude of as much as 0.4 dB are revealed over the first few hundred microseconds after turn-on. At the same time, this phase-sensitive measurement reveals phase drift behaviors: for all devices tested, a fast phase shift of several radians occurs over the first several tens to hundreds of microseconds, followed by a smaller drift over millisecond timescales. During these tests, the observed switching time is limited by the 25 MHz measurement bandwidth of our digitization hardware, but relevant phase and amplitude fluctuations over milliseconds are readily apparent. The magnitude of both amplitude and phase shifts increase as drive current is increased, and are understood to be due to thermalization as current is rapidly turned on. While these phase shifts do not adversely affect the MOT-based atom cooling scheme, relative phase shifts may present issues for some functions, such as atom transport and phase-sensitive interferometry. Fiber-optic phase fluctuations due to thermal drift can easily be of the same magnitude, and should also be suppressed in such systems. While cancellation of SOA-induced phase fluctuations to some level should be achievable based on a thermal pre-emphasis approach via tuning of each chip’s TEC current, closed-loop electro-optic control may nonetheless be required to cancel all sources of phase drift prior to the physics package in future systems.

### Environmental sensitivity

Throughout tests, all SOAs exhibited consistent switching dynamics at short time-scales, indicating the potential for highly repeatable operation in an all-semiconductor system. However, high-fidelity measurements with cold-atom quantum sensors requires stability over both shot-to-shot and longer measurement-relevant time-scales, since fluctuations in laser power can translate to instrument noise.

**Figure 4:**
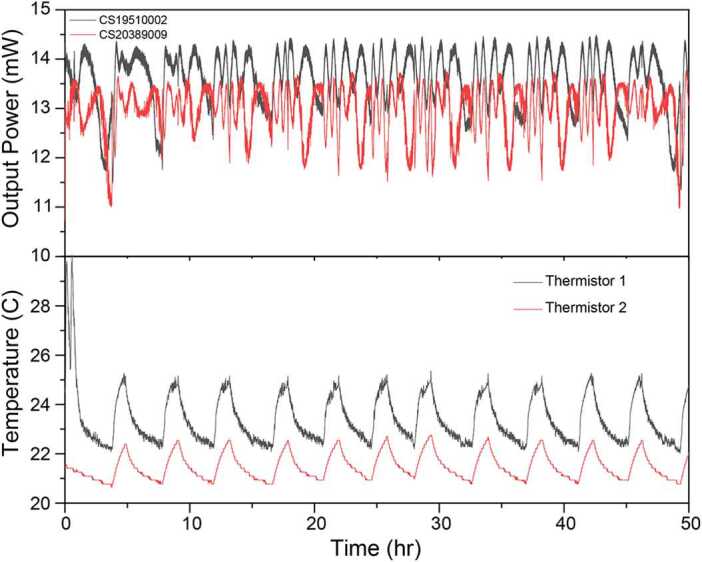
Output power fluctuations for two 852 nm SOA devices over a period of 50 hours after turn-on. As the room temperature changes due to climate control, the SOA transmission fluctuates in a fringing pattern due to thermal gradients.

While operating initial versions of our prototype LOS, we observed that the SOAs suffered from relatively large fluctuations in output power over s-hr timescales. In fact, observed output power fluctuations were significant across all tested devices: as much as 18% for the 852 nm SOAs used for Cs atom cooling (Superlum SOA-372-DBUT-PM), and around 4% for an array of other devices at 780 nm and 852 nm (Innolume SOA-780-20-YY-30dB, Thorlabs BOA780P, Superlum B850.30.40P) including an 852 nm tapered amplifier which used bulk optics to facilitate fiber coupling (MOGLabs MOA-L). Example data for two 852 nm SOAs over a couple of days is plotted in Fig. 4. Although all chips were mounted on tightly-controlled thermoelectric coolers (TECs) within butterfly packages, the observed output power fluctuations clearly correlate with changes in room temperature as measured from thermistors mounted on the driver housing (1) and in the same room but away from the active device (2). Shorter term fluctuations on second-level scales were also observed, particularly when room doors were opened or operators were present. Further investigation with local heaters revealed that thermal gradients across the package, an in particular in the vicinity of coupling fiber optics, led to rapid, large power swings. The particular sensitivity of chip-based amplifier systems is perhaps not surprising: compared to semiconductor lasers which require only output coupling, both fluctuations in the input and output coupling ratio are compounded in SOAs. By comparison, when the same 852 nm SOAs were not seeded, the ASE light exiting each port was stable to within 1% over the same time scales.

For future science-grade LOS architectures, shot-to-shot and long-term power stability should be much better than the 1% level, suggesting the need for some form of active power monitoring and control to meet future power stability requirements. We anticipate that this will be true both for systems that use high power laser amplifiers followed by signal distribution (e.g. conventional MOPA configurations) as well as SOA-based architectures, since all of these experience output power drift over time.

### Active power stabilization

To experimentally validate the capability for power stabilization via current feedback to SOAs, we inserted inline photodiodes (OZ Optics OPM-11-850) after each SOA output as shown in Fig. 2b, which tap a ratio (2%) of light to measure the true output power. Photocurrents are read out using the same main control board, and closed-loop feedback for each channel is then implemented in microcontroller firmware.

For a continuous-wave output, high-fidelity closed-loop control is straightforward to implement since power data are continuously acquired. However, in our architecture, the output power of the SOA is rapidly switched and tuned during the preparation and measurement sequence for the atomic ensemble. To simulate this situation, we implement a notional power sequence consisting of several abrupt changes in output power including a brief (ms) pulse. Measured optical power from earlier sequences or earlier steps within the same sequence is then used to adjust SOA drive current; power is not adjusted within an individual step, since intra-step stability is prioritized over accuracy and the duration of the shortest steps is comparable to filtering lags in the photodiode readout.

In its simplest incarnation, each individual SOA power step is treated independently and is adjusted linearly based on that step’s deviation from ideal in the previous sequence; this performs well for stable SOAs, but the lag from the previous sequence (on the order of a second for a typical system operating at Hz-level measurement rates) poses a challenge for suppressing rapid transients. Better performance is achieved by inserting two or more calibration steps into the sequence in a dead period where output power is not critical (or is directed away from the physics package), using these measurements in firmware to constraint a simple model for the SOA, and using that model to perform the sequencing.

**Figure 5:**
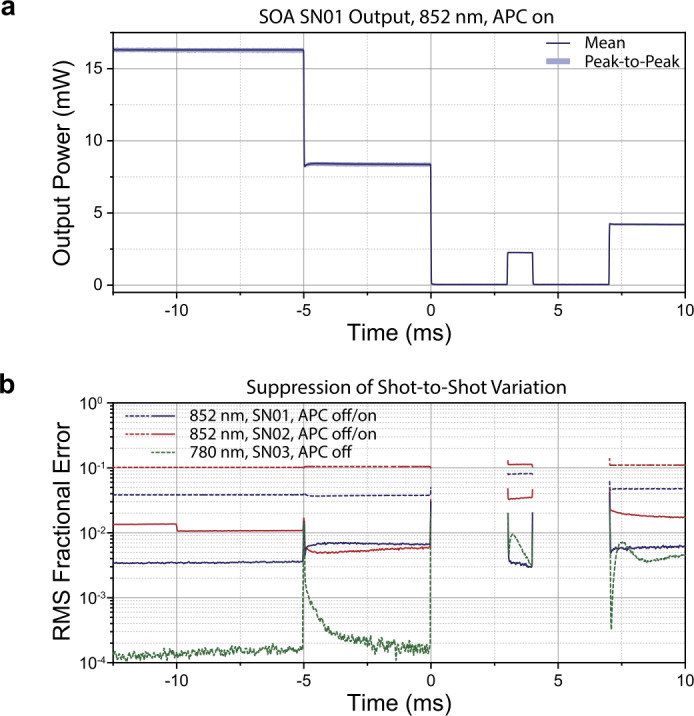
Demonstration of power modulation and active power control (APC). (a) A nominal power sequence is implemented via current switching of the SOA bank. Each SOA channel includes an inline photodiode mounted in the same control board. To improve output power stability, photocurrent readout and feedback to SOA drive current is implemented with digital control electronics. The resulting output power is measured using an external photodiode for accuracy. Statistics are acquired over 300 pulse sequences. (b) plots a comparison of power stablity of three devices with active power control switched on and/or off. The free-running stability of the 780 nm device (SN03), was much better than the 852 nm devices, and was only substantially improved with APC turned on beyond >1 minute timescales.

Results for an abbreviated notional sequence that is repeated at a rate of 1 Hz are plotted in Fig. 5a-b. Compared to the observed fluctuations of up to 18% for the free-running SOAs, with active power control enabled power fluctuations are reduced to around 1% RMS for most of the sequence duration, representing an average improvement of over 10×. The ultimate performance of this method is highly dependent on the SOA drift properties and the proximity in time of the critical sequence steps to the calibration steps. However, improvements to the SOA stability by improved engineering of input/output coupling will directly translate to further enhancements to overall stability. For comparison, the short-time behavior of one of the 780 nm SOAs tested (Innolume SOA-780-20-YY-30dB) is plotted in green in Fig. 5b, and already reaches the 1% level. In fact, the stability of this SOA is not significantly improved by the current active feedback mechanism except at >1 minute timescales, where all tested amplifier devices exhibited substantial power drift. Throughout these measurements, the inline photodiodes measure the total power output from the SOAs–separate heterodyne measurements with the method shown in Fig. 3a indicate that the relative powers of multi-tone signals (e.g. pump and repump) are maintained through operation. With further improvements to the feedback algorithm, we anticipate stability of better than 0.1% RMS shot-to-shot can be achieved. Nonetheless, these results show the feasibility of parallelized active power stabilization across numerous output channels simply by leveraging intrinsic current-tuning of the SOA gain, and without the need for additional modulator components.

### Added noise

**Figure 6:**
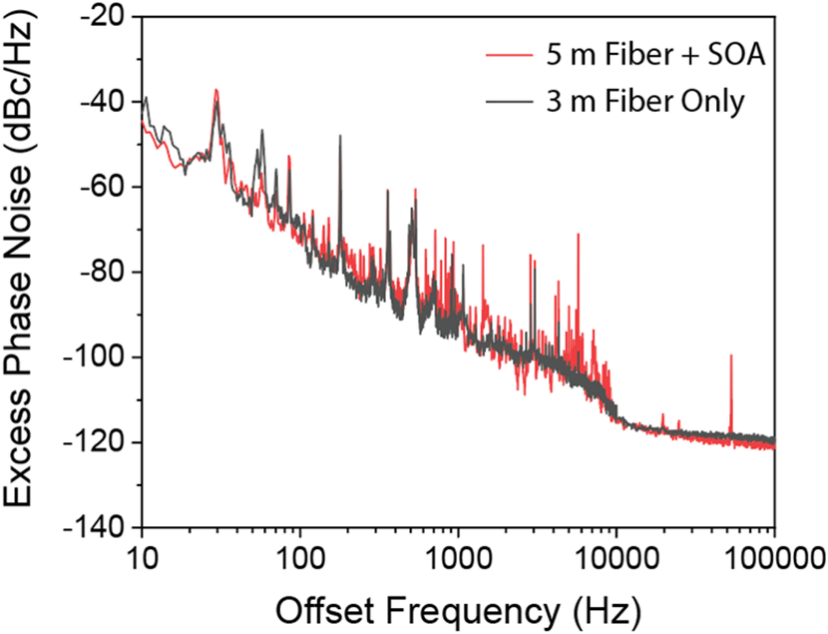
Excess phase noise between two arms of an imbalanced Mach-Zehnder interferometer with either a 3 m differential fiber path difference (grey) or a 5 m differential fiber path difference with the signal amplified using an SOA.

Future in-orbit atom interferometry operating over interrogation times of tens of seconds will have extremely stringent requirements on spectral purity of the laser beams used for atom manipulation. SOAs are generally understood to not impact laser linewidth (phase noise level), which can be tested by measuring laser linewidths before and after amplification. However, in the future, sophisticated forms of laser cooling and control of atoms may require extremely narrow linewidth (sub-kHz) laser signals, and thus will be sensitive to technical phase noise arising from external optics, including vibration-induced noise. To briefly examine whether any additional noise could be observed as arising from the use of an SOA, we utilized the same heterodyne measurement as in Fig. 3a to estimate the phase noise added between the two interferometer arms with or without a SOA present and powered on. The measured excess phase noise under these conditions is plotted in Fig. 6. In both situations, some noise is observed, particularly below 10 kHz offset frequencies where fiber microphonics contribute substantial phase fluctuations between the two arms. When the SOA is introduced, this measured fiber phase noise is unchanged, except for a few electrical peaks (e.g. 53 kHz) likely arising from the drive electronics. These results corroborate the conventional notion that SOAs do not impact laser phase noise, at least to the ∼Hz linewidth level limited by the sensitivity of this measurement technique.

Like all amplifiers, SOAs also add some additional broadband noise in the form of amplified spontaneous emission. While ASE over ∼10 s of nanometers bandwidth was observed at low seed powers, for all SOA devices tested this ASE level was seen to decrease with increasing seed power, to the point where it was not measurable within the dynamic range of a commercial optical spectrum analyzer (Thorlabs OSA201C) under typical operation conditions of our prototype LOS (seed power >50 μW, output power >10 mW). For ∼20 mW/channel output powers used for atom cooling, these initial measurements constrain the ASE level around the carrier to a spectral density of <1 μW/nm.

## Discussion

We characterized the performance of commercial semiconductor optical amplifiers for use as both high-extinction-ratio switches and power boosters/modulators and implemented them within a proof-of-concept Cs-atom cooling laser and optics system. This approach can potentially reduce the need for power-hungry acousto-optic components commonly used for high-performance switching and power control, while offering superior performance as compared to electro-optic or mechanical switches. In our SOA-based system, all necessary functionalities for atom cooling were achieved without the need for high-power lasers or amplifiers, reducing total supply power requirements while offering potential benefits to system reliability.

Another attractive feature of the all-semiconductor laser and amplifier approach to LOS design is that it can be adapted to atomic species requiring very different operation wavelengths. SOAs are available across a wide range of wavelengths relevant to atomic physics (down to as short as 400 nm) [36, 37], and use similar material technologies and design concepts to semiconductor lasers that are already an essential element to most cold-atom quantum sensor systems [38–40]. As a result, utilizing SOAs for switching and amplification in these systems reduces the number of distinct material platforms that need to be qualified for operation in the relevant flight or space environment. At the same time, using moderate-power SOAs at the output of the laser and optical system is inherently more power efficient than using higher-power amplifiers upstream, where laser signals accumulate optical losses as they traverse the numerous optical components prior to collimators at the input of the atom physics package.

Generally speaking, a shortcoming of existing commercial SOAs at relevant wavelengths for atom cooling is the relatively low available output powers–below 100 mW at 780 nm and less than 50 mW at 850 nm, limited by potential catastrophic optical damage at the output facet [38]. This limitation is not fundamental, since waveguide cross-sections can be increased to reduce optical power density; however, it seems that commercial parts are optimized for applications where high gain but relatively low powers are needed. While these powers are sufficient for many operations, the development of an atom interferometry-capable LOS will require higher powers for interferometry pulses and transport beams. Tapered amplifiers are commonly employed to this end, and represent an alternate implementation of identical physics to SOAs–the primary difference being that the output waveguide is gradually tapered up in width, resulting in high power handling at the expense of a highly astigmatic beam. Most commercial TAs are optimized for extremely high output power of 1-5 W, which requires drive currents of several amperes and sophisticated thermal management, and corresponding high power consumption. The design and fabrication of SOAs designed for intermediate power operation in the 100-200 mW range would represent a highly beneficial technology for future low-SWaP laser systems for atom interferometry, both by increasing the power budget available for various cooling stages, and by potentially allowing direct synthesis of high power pulses (e.g. for Raman pulse or Bragg pulse interfeormetry). This could be achieved by developing chips which use larger optical mode sizes, or through alternate device designs such as slab-coupled amplifiers [41].

Through our tests, we evaluated a number of fiber-coupled SOA and TA chips and found that all exhibited substantial environmental sensitivity, particularly to thermal gradients. As a result, closed-loop power control seems likely to be necessary for all such laser systems, including conventional MOPA-based architectures that use high-power TAs. To address this issue, we implemented scalable power monitoring and digital feedback in each output channel using inline photodiodes, achieving a 10× improvement in stability even during pulsed operation with rapidly-varying power levels. Engineering improvements for SOA fiber coupling and improvements to control algorithms will result in further improvements to achievable power stability.

In parallel with the development of a prototype all-semiconductor laser and amplifier LOS, we are currently investigating the long term reliability of SOAs under pulsed excitation. Initial results (Appendix C) reveal no obvious deleterious effects due to current pulsing over a few thousand hours, although further detailed lifetime testing of multiple devices is needed. Prior literature of SOA reliability suggests the potential for lifetimes exceeding 10^5^ hr (>10 years), with similar aging mechanisms to semiconductor lasers [42–44]. These preliminary tests are promising for the space qualification of SOAs based on similar technology to existing semiconductor lasers routinely operated in space.

## Conclusion

We have evaluated the performance of semiconductor optical amplifiers to act as switches, modulators, and power boosters, and constructed a self-contained and scalable SOA-based laser system for cooling a cloud of neutral cesium atoms. High extinction ratio switching and power-efficient operation were demonstrated, while potential shortcomings were investigated. In order to compensate for output power fluctuations arising from environmental effects, we implemented automatic power monitoring and closed-loop current feedback to improve power stability even during pulsed operation. Including custom path-to-flight drive and locking electronics, the maximum DC power consumption of the prototype system was around 13.5 W with all SOAs energized. The all-semiconductor approach represents an alternative path to laser system design that reduces the need for power-hungry high-power lasers and acousto-optic switches, and may facilitate the maturation of cold-atom quantum sensors into field- or space-deployable instruments.

## Figures and Tables

**Figure 7:**
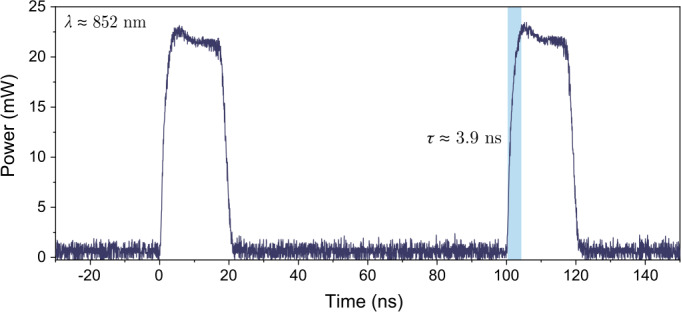
Measurement of ∼20 ns optical pulses generated through an SOA resulting from square wave current pulses.

**Figure 8:**
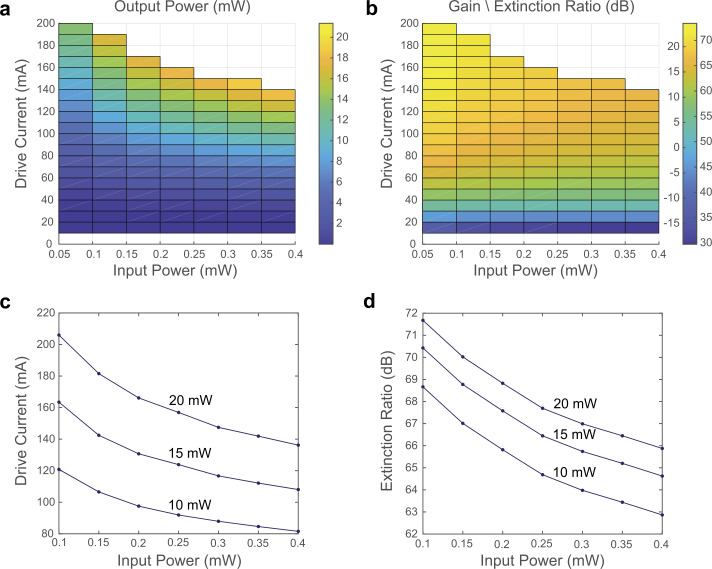
Gain and on-off extinction ratio for one SOA module at 852 nm. (a) plots output power as a function of input power and electric current for one low-power device. (b) plots total gain and extinction ratio under the same conditions (gain and ER are denoted by the left and right labels on the color bar, respectively). (c) and (d) plot the required drive current and demonstrated extinction ratio for output power levels of 10, 15, and 20 mW as a function of input power, respectively. >60 dB on/off extinction ratio is achieved across a wide range of conditions at these output powers. Similar results were measured for other devices at 852 nm and 780 nm.

**Figure 9:**
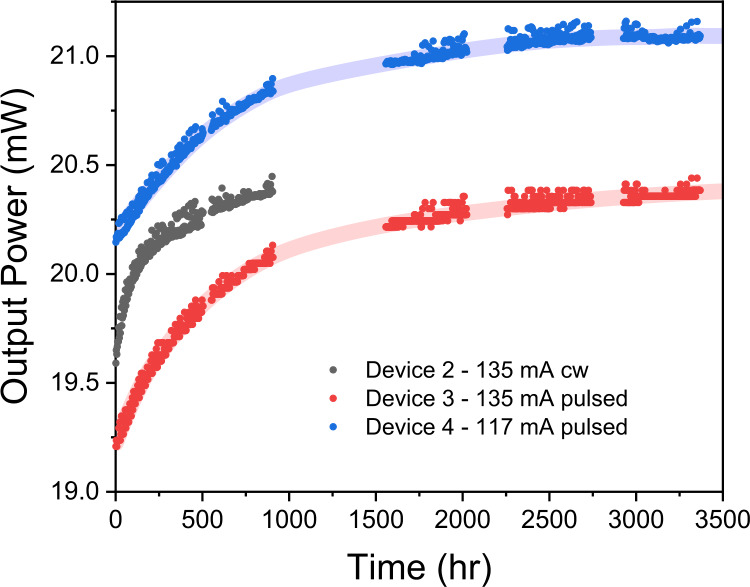
Long-term testing of a few 852 nm SOA devices. Three commercial SOA devices (Superlum SOA-372-DBUT-PM) from the same fresh batch were seeded by the same laser and their output power was recorded over time. While long-term aging is expected to decrease gain and output power, these devices exhibited the opposite behavior. Nonetheless, pulsed operation of SOAs did not seem to adversely affect behavior over relatively short timescales of a few thousand hours. While the devices were operated continously, there are some gaps in data acquisition–shaded regions are guides for the eyes.

## Data Availability

No datasets were generated or analysed during the current study.

## Appendix A: Switching speed

Although nanosecond switching speeds are not necessary for atom cooling applications, we briefly investigated the speed of the SOA devices under test. For this measurement 20-ns-long, 140 mA current pulses are synthesized using a commercial pulsed driver (Aerodiode SOA-STD) and drive a temperature-stabilized SOA (Superlum SOA-372-DBUT-PM) seeded by 0.2 mW of light from an external DBR laser (Photodigm). The output light is incident on a fast photodiode (Discovery Semiconducor DSC-R402AC-SW-39) and digitized using an oscilloscope (Tektronix DPO72004DX). The result, shown in Fig. 7, is a corresponding series of optical pulses with an on/off switching time of around 3.9 ns, comparable to prior measurements of SOA speed [45, 46]. An overshoot in output power occurs over the first 10 ns, possibly due to the release of stored energy as current is ramped up, which could be mitigated via pre-emphasis or slower ramping of current speed if required. More sophisticated pulse shaping should also be possible via current modulaton, since the measured switching speed far exceeds the >μs rates required of most practical cold-atom systems.

## Appendix B: Extinction ratio and gain

To establish a baseline for device performance, we conducted basic quasi-continuous wave (quasi-cw) testing of devices to evaluate gain, output power, and on/off extinction ratio, as shown for one 852 nm SOA in Fig. 8. To avoid potential optical damage at the output facet, throughout these tests output power was limited to less than 20 mW (852 nm). Input seed light in each case is provided from commercial distributed Bragg reflector (DBR) lasers (Photodigm) with a variable attenuator, while high dynamic range power measurements are made using a fiber-optic photodiode power meter (Thorlabs PM102A/S150C). In the absence of applied current (‘off’ state), incident light is strongly absorbed–the measured absorption was independent of input power over the tested range and was −49 dB (852 nm) and −47 dB (780 nm). When drive current is turned on, incident light experiences gain–the on/off extinction ratio is identically the net gain minus the on/off absorption, and so both can be plotted using a single scale as in Fig. 8b. The required drive current to achieve certain output power levels is interpolated from these panels a-b and is plotted in Fig. 8c, while the resulting on/off extinction ratio under these conditions is plotted in Fig. 8d. Notably, very high extinction >60 dB is maintained across typical conditions, indicating that SOAs can be useful for high-extinction-ratio switching across an array of operating parameters. These tests were repeated for other devices of the same part number, which displayed comparable gain and extinction ratio performance to within ∼3 dB. For devices operating at 780 nm, output power was limited to less than 70 mW and similar gain and extinction ratio numbers were observed.

These results establish the basic operation of SOAs as both power boosters and high-extinction-ratio switches. By measuring the gain curve vs. current for a specific input power, output light intensity may be controlled for pulse shaping, power stabilization, or optical modulation.

## Appendix C: Long-term operation of pulsed SOA devices

In parallel with the system development and SOA characterization efforts, we sought to perform initial tests of device aging to evaluate whether continuous current pulsing adversely affects their reliability. To date, prior studies of SOAs under continous-wave operation have reported aging to be relatively gradual, involving a slow decrease in gain over time accompanied by a proportional decrease in ASE power under unseeded operation. This decrease in gain is understood to arise from the decrease in carrier density with aging, resulting from diffusion of non-radiative recombination centers. Lifetimes reported for SOAs in the literature can exceed 10^5^ hours (>10 years) continuous operation at room temperature and moderate current densities [42–44]. However, the extent of prior literature studies of SOA aging is relatively limited, and these studies tend to focus on continuous-wave ‘on’ operation.

For our initial tests, we seeded three nominally identical SOA devices from the same laser delivering ∼0.45 mW input power to each amplifier, and constantly measured their output power over several thousand hours. Two devices, ‘Device 3’ and ‘Device 4’ were driven by 3-second current pulses with a 50% duty cycle (135 mA and 117 mA, respectively), while a third device ‘Device 2’ was driven be a constant 135 mA current. These drive currents were chosen so that all devices had roughly equal output powers around 20 mW despite some difference in measured gain between the three devices. Due to output power fluctuations, periodic measurements of ASE (seed laser off) were used to calibrate environmentally-sensitive input and output coupling ratios, and averaging was used to produce the data in Fig. 9. Counter-intuitively, all devices exhibited an *increase* in gain and output power over time, perhaps revealing a form of burn-in for these specific devices. This aging occurred more rapidly for the cw device ‘Device 2’, indicating that it may be proportional to average current density. Unfortunately, due to a limited number of devices available for LOS construction, Device 2 was removed after around 1000 hours and instead used in the LOS demonstration, while the pulsed devices were continually operated for several thousand hours. Throughout these tests, opacity of the pulsed SOAs was not observed to decrease over time, so that the same high-extinction-ratio switching was maintained.

At least for operation under laboratory conditions, these results suggest that pulsing does not adversely affect device lifetime. However, they are confounded by the exhibited burn-in as well as limited number of devices under test. Looking forward, greater numbers of devices can be used for rigorous pulsed lifetime testing via accelerated aging measurements: Both increased temperature and increased current density are well-known to accelerate aging for both laser and SOA devices. The temperature dependence is similar to that observed in laser devices, and is mathematically related to an empirical ‘activation energy’ through an Arrhenius equation that can be understood as modelling the temperature dependence of diffusion coefficient. The current-density-induced aging may also be related to local heating. Using this approach, operation at a series of successive temperature steps and measurement of output power or ASE power over time can be used to estimate a lifetime for each individual device–repeating across many devices can thereby infer population statistics.
